# Biphasic Modulation of NOS Expression, Protein and Nitrite Products by Hydroxocobalamin Underlies Its Protective Effect in Endotoxemic Shock: Downstream Regulation of COX-2, IL-1**β**, TNF-**α**, IL-6, and HMGB1 Expression

**DOI:** 10.1155/2013/741804

**Published:** 2013-05-28

**Authors:** André L. F. Sampaio, Jesmond Dalli, Vincenzo Brancaleone, Fulvio D'Acquisto, Mauro Perretti, Carmen Wheatley

**Affiliations:** ^1^The William Harvey Institute, Barts and The London School of Medicine and Dentistry, Queen Mary University of London, Charterhouse Square, London EC1M 6BQ, UK; ^2^Far Manguinhos—FIOCRUZ, R. Sizenando Nabuco 100, 21041-250 Rio de Janeiro, RJ, Brazil; ^3^Department of Science, University of Basilicata, Potenza, Italy; ^4^Orthomolecular Oncology, Registered Charity No. 1078066, 4 Richmond Road, Oxford OX1 2JJ, UK; ^5^St Catherine's College, Oxford University, Manor Road, Oxford OX1 3UJ, UK

## Abstract

*Background*. NOS/^•^NO inhibitors are potential therapeutics for sepsis, yet they increase clinical mortality. However, there has been no *in vivo* investigation of the (*in vitro*) ^•^NO scavenger, cobalamin's (Cbl) endogenous effects on NOS/^•^NO/inflammatory mediators during the immune response to sepsis. *Methods*. We used quantitative polymerase chain reaction (qPCR), ELISA, Western blot, and NOS Griess assays, in a C57BL/6 mouse, acute endotoxaemia model. *Results*. During the immune response, pro-inflammatory phase, parenteral hydroxocobalamin (HOCbl) treatment partially inhibits hepatic, but not lung, iNOS mRNA and promotes lung eNOS mRNA, but attenuates the LPS hepatic rise in eNOS mRNA, whilst paradoxically promoting high iNOS/eNOS protein translation, but relatively moderate ^•^NO production. HOCbl/NOS/^•^NO regulation is reciprocally associated with lower 4 h expression of TNF-*α*, IL-1**β**, COX-2, and lower circulating TNF-*α*, but not IL-6. In resolution, 24 h after LPS, HOCbl completely abrogates a major late mediator of sepsis mortality, high mobility group box 1 (HMGB1) mRNA, inhibits iNOS mRNA, and attenuates LPS-induced hepatic inhibition of eNOS mRNA, whilst showing increased, but still moderate, NOS activity, relative to LPS only. experiments (LPS+D-Galactosamine) HOCbl afforded significant, dose-dependent protection in mice *Conclusions*. HOCbl produces a complex, time- and organ-dependent, *selective* regulation of NOS/^•^NO during endotoxaemia, corollary regulation of downstream inflammatory mediators, and increased survival. This merits clinical evaluation.

## 1. Introduction

**Figure 1:**
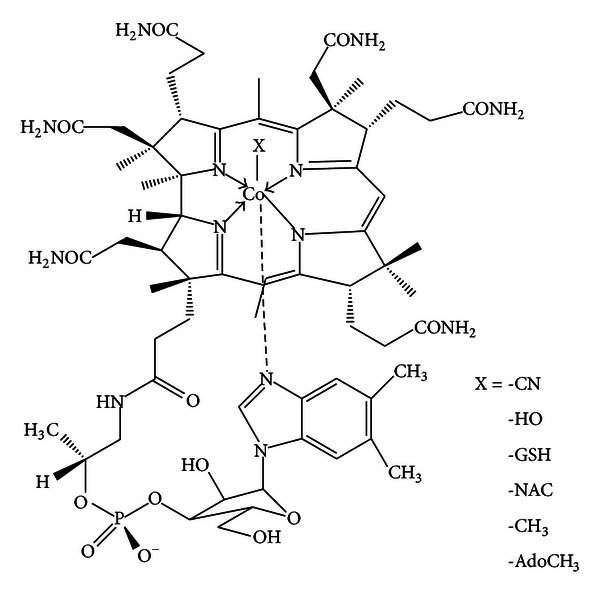
The structure of cobalamin. X = the principal ligands for the cobalt atom, in the upper, *β* axial position.

Cobalamin, C_63_H_88_O_14_N_14_PCo (Figure 1), participates in only two known mammalian enzymatic reactions. Yet, these two Cbl-dependent enzymes, cytosolic methionine synthase (MS) [EC 2.1.1.13], requiring methylcobalamin (MeCbl), and mitochondrial methylmalonyl-CoA mutase (MU) [EC 5.4.99.2], requiring adenosylcobalamin (AdoCbl) [1, 2], are critically involved in key metabolic pathways essential for gene expression and regulation, *via* formation of S-adenosylmethionine (SAM) and methylation, and in protein synthesis and catabolism, cellular respiration, and energy. Activation of methionine synthase also ensures key antioxidant defense status, as it triggers concurrent activation of cystathionine *β*-synthase (C*β*S), the pivotal enzyme at the homocysteine junction in the trans-sulfuration pathway to glutathione (GSH) [3].

Cobalamin is the standard treatment for autoimmune “pernicious” anaemia, and macrocytic or megaloblastic anaemia, as well as for subacute combined degeneration of the spinal cord. However, an increasing body of work suggests that Cbl may also play a central role in the regulation of immunity and inflammation (reviewed in [4]). Cbl confers significant protection in various animal models of shock, from anaphylaxis to trauma and sepsis [5–7], and has remarkable organ/tissue protective effects when used clinically for the treatment of analogous inflammation in CN poisoning (reviewed in [8]). Amongst Cbl's known immunological effects are an augmentation of the CD8+/CD4+ T-lymphocyte ratio and natural killer cell activity [9, 10], both significantly reduced in inflammatory pathology, with negative consequences in septic patients [11].

Interesting homeostatic links between Cbl and pivotal cytokines are also emerging, indicative of complex but still incompletely defined regulatory circuits: MeCbl lowers interleukin-6 (IL-6) expression in peripheral blood monocytes [12], whilst Cbl deficiency raises circulating IL-6 in humans [13] and Cbl physiological status regulates IL-6 levels in rat cerebrospinal fluid [14]. Moreover, in both rodents and humans there appears to be an inverse relation between Cbl physiological levels and tumour necrosis factor alpha (TNF-*α*) serum levels [15]. *In vitro*, neuronal Cbl deficiency is also associated with increased expression of two TNF-*α*-converting enzyme secretases [16]. A reasonable hypothesis is that such Cbl/TNF-*α*/IL-6 regulation may be partly effected *via* Cbl indirect regulation of the central immune regulatory transcription factor, nuclear factor kappa B (NF-*κ*B) [8]. Normal physiological levels of Cbl in spinal fluid appear to correlate with NF-*κ*B quiescence, at least, in a non-inflammatory/non-immune challenge model [17]. Recently, a kinetic study reported that Cob(II)alamin reacts with superoxide at rates approaching superoxide dismutase [18]. CNCbl protects human aortic endothelial cells, and neuronal cells, *in vitro, *against superoxide induced injury [19, 20]. Given that oxidative stress is a major trigger of NF-*κ*B activation, this potential antioxidant effect of Cbl could theoretically lead to NF-*κ*B inhibition. It may also be of critical local importance *in vivo*, as the phagocytic burst includes release of the Cbl carrier, haptocorrin (HC/TC 3), in the immediate vicinity of NADPH oxidase [8, 21] one of the major biochemical sources of superoxide in immune challenge and inflammation [22]. HC/TC3, moreover, is upregulated by IL-1*β*, itself expressed within fifteen minutes of inflammatory challenge [23]. Nevertheless, though antioxidant effects of Cbls have been observed *in vitro* [24] and may, indeed, be important *in vivo* [20], no systematic analysis of the *in vivo* mechanisms of Cbl conferred protection against inflammation during *acute* immune challenge has hitherto been done.

We wondered if a more comprehensive explanation for Cbl effects on inflammation and immunity, and thence beneficial outcomes in sepsis and other forms of shock, may lie in a potential direct/indirect regulation by Cbl of one or more of the several actions of nitric oxide (^•^NO) as a ubiquitous, cell-signal transduction molecule and second messenger for post-translational modification, whose targets include soluble guanylate cyclase [25].^•^NO is the product of three nitric oxide synthases (NOS): two constitutive, nNOS (neuronal NOS; NOS I) and eNOS (endothelial NOS; NOS III), and one inducible, iNOS (NOS II), at much higher levels of expression, with the potential to produce 1000-fold higher than normal amounts of ^•^NO, during gestation, growth, and the immune response [26]. Cobalamins are known to have effects on ^•^NO [27–29], but these have hitherto been thought to be a consequence of Cbl/^•^NO scavenging effects [7, 30–35] demonstrable chemically and *in vitro* [36, 37], but biologically unproven *in vivo* and still controversial [38–40].

Nitrosylcobalamin has not been detected, to date, *in vivo *or *in vitro, *amongst naturally occurring intracellular Cbls [41]. Nevertheless, if the hypothesis that Cbl is involved in NOS catalysis has any substance [42, 43], then it is conceivable that, in analogy to previously observed ferric-heme-NO complex formation at the conclusion of NOS catalysis [44], NOCbl might be transiently formed, just prior to release of free ^•^NO by the NOS [43]. Such a theoretical transience and discrete localisation might account for the failure to detect NOCbl *in vivo *to date. Ubiquitous and continuous Cbl scavenging of ^•^NO, on the other hand, may pose biochemical hazards. For ^•^NO has important antioxidant and cell-signalling actions [25] which might be obstructed by HOCbl's previously proposed, indiscriminate ^•^NO scavenging, or even just by a recently proposed, Cbl structural-based, direct inhibition of the NOS *tout court* and nothing else [45]. There is some evidence that HOCbl can discriminate between exogenous ^•^NO donors and the natural endogenous donor, *S*-nitrosoglutathione, GSNO, actually prolonging only GSNO-induced, gastric fundus relaxations [46]. This hints at a more complex Cbl/^•^NO regulatory relationship.

Moreover, there are also diverse indications that positive Cbl status is allied to beneficial ^•^NO activity: in diabetic rats, high cobalamin levels correlate with high NOS protein levels, ^•^NO activity, and increased erectile function [47]; Cbl supplementation of vegetarians with low Cbl status significantly increases eNOS ^•^NO release in the brachial artery [48]; in the digestive tract of endotoxemic rats, the highest expression of iNOS is in the ileum, precisely where Cbl is internalized [49], and both Cbl and ^•^NO are known to mediate cell protective effects via ERK1/2 and Akt [50–54]. These protective effects of ^•^NO and Cbl include induction and regulation of heme oxygenase-1 (HO-1) [52, 55–58], which converts biliverdin to the powerful antioxidant bilirubin, and carbon monoxide. CO can then in turn decrease ^•^NO [59]. (For a more comprehensive list of coincidences of Cbl's/^•^NO's positive actions, see Table 1 and its related discussion in [43]).

Thus, in these studies we explored an alternative hypothesis to that of Cbl as *just* an ^•^NO, or, indeed, superoxide, mop. We posited that the principal mechanism behind Cbl's beneficial, pleiotropic effects in inflammation may involve a biphasic regulation of NOS expression and protein translation and the ensuing ^•^NO synthesis, during the two distinct pro- and anti-inflammatory phases of the immune response.

## 2. Materials and Methods

### 2.1. Animals

Male C57BL/6 mice, weighing 20 to 25 g, were purchased from Harlan, UK, and maintained on a standard chow pellet diet, containing standard amounts of Cbl (50 *µ*g/kg vitamin B12/CNCbl), with tap water supplied *ad libitum*. Animals were kept in a 12:00 h light/dark cycle, and all were housed for 7 days prior to experimentation. All experiments were performed in accordance with UK Home Office regulations (Guidance On the Operation of Animals: Scientific Procedures Act, 1986).

### 2.2. Cobalamins

The coenzymes, 5′-deoxyadenosylcobalamin, and methylcobalamin; Vitamin B_12_a, cyanocobalamin, and hydroxocobalamin (CAS 78091-12-0) were purchased from Sigma-Aldrich (UK). Glutathionylcobalamin and N-acetyl-cysteinyl-cobalamin were synthesized and supplied by Professor Nicola Brasch (Kent State University, Ohio, USA). All Cbls (and Cbl-treated animal samples) were protected from light during storage and handling, and were 98% to 99.5% pure.

### 2.3. Drug Treatment and Experimental Design

5′-deoxyadenosylcobalamin (AdoCbl), methylcobalamin (MeCbl), hydroxocobalamin (HOCbl), glutathionylcobalamin (GSCbl), and N-acetyl-cysteinyl-cobalamin (NAC-Cbl) were all stored at −20°C, and fresh solutions of them were made using sterile, pyrogen-free, phosphate-buffered saline (PBS; Gibco), prior to the experiments. For the *in vivo* experiments, cobalamins were diluted at 10 mL/kg prior to treatments (with PBS used as vehicle). Cobalamins were administered according to the protocol summarized in Table 1.

### 2.4. Effects of Endogenous Cobalamins on NF-*κ*B Promoter Activity

RAW 246.7 macrophage cells, stably transfected with NF-*κ*B luciferase reporter construct (Stratagene), were maintained in Dulbecco's modified Eagle's medium, supplemented with 10% (v/v) fetal bovine serum, 2 mM l-glutamine, 1 *µ*g/mL Geneticin, and 50 *µ*g/mL G418. Cells (2 × 10^4^ cells) were seeded in 96-well plates and then preincubated for 1 h with increasing concentrations (1–10–100 *µ*M) of the five principally occurring, intracellular Cbls. Thereafter, at time 0 h, cells were stimulated with *E. coli* LPS (0111 : B4; 1 *µ*g) for 4 h and then processed for measurement of luciferase activity in a luminometer (Luminometer TD-20/20; Turner Designs Instruments).

### 2.5. Non-Lethal and Lethal Endotoxaemia

Endotoxaemia was induced by the intraperitoneal injection of LPS (0.1 mg/kg), alone (non-lethal) or, in the lethal endotoxaemia protocol, in combination with 1 g/kg D-Galactosamine (Table 1). Sample collection in non-lethal endotoxaemia was carried out at both 4 and 24 h after LPS challenge. Animal survival, in all lethal endotoxaemia experiments, was monitored for a total of 5 days, and all data were analysed using Chi-squared or Kaplan-Meier tests.

Times shown are in relation to time 0 h, when either LPS alone or LPS+D-Gal was administered by intraperitoneal injection. Individual cobalamins were injected into the peritoneum at the doses and times reported in Table 1.

### 2.6. Sample Preparation for Real-Time Reverse Transcriptase-PCR

Blood (500 *µ*L) was centrifuged (for 5 min, at 2500 rpm), and the plasma then collected for ELISA analysis. 500 *µ*L of TRIZOL reagent (Invitrogen) was added to the remaining fraction. RNA purification was performed as recommended by the manufacturer. Following extraction, RNA (20 *µ*L) was treated with 2 U (1 *µ*L) of TURBO DNase 1 (Ambion, Austin, TX), as described by the manufacturer, to remove any contaminating genomic DNA. An aliquot of the DNA-free RNA (7.6 *µ*L) was then transferred to a new RNase-free tube and reverse-transcribed into complementary DNA (cDNA), using Superscript III Reverse Transcriptase (Invitrogen), as described by manufacturer. The following reagents were used: Oligo dT primers (Invitrogen); 1 *µ*L, 10 mM dNTP (Bioline); 4 *µ*L of 5X first-strand buffer; 1 *µ*L, 0.1 M DTT; 1 *µ*L (40 U) RNaseOUT; and 1 *µ*L (200 U) of Superscript III Reverse Transcriptase (Invitrogen). After synthesis, cDNA was quantified using a Nanodrop ND-1000 and diluted (80 ng/*µ*L) in molecular biology grade water and then loaded into 384-well plates for real-time PCR.

### 2.7. Real-Time Reverse Transcriptase PCR

Real-time PCR assays were performed on the various samples in order to evaluate the expression of the following genes: GAPDH, RPL32, IL-1*β*, COX-2, iNOS, eNOS, TNF-*α*, and HMGB1 (Table 2). For each gene analyzed, reactions were performed using 1 *µ*L of the Qiagen QuantiTect Primer Assay, added to 5 *µ*L Power SyBR Green PCR Master Mix (Applied Biosystems, Warrington, UK) and then diluted with 2 *µ*L molecular grade water. A final volume of 8 *µ*L was dispensed into each well and 2 *µ*L of diluted cDNA (160 ng/reaction) was added. Each sample was tested in triplicate for each gene, and PCR reactions were performed using ABI Prism 7900 real-time PCR equipment. The thermal profile consisted of 95°C for 15 min, then 40 cycles of 94°C for 15 s, 55°C for 30 s, and 72°C for 30 s. This was plotted as a melting curve. The comparison between samples was performed using GAPDH and RPL32 as internal standards. REST MCS software was utilized for the calculation of the relative difference between the test groups.

### 2.8. iNOS and eNOS Western Blotting

Liver tissues were harvested from (*n* = 5) animals, after LPS endotoxaemia, with or without HOCbl treatment and then homogenized in lysis buffer, which contained a cocktail of protease inhibitors. Protein concentrations prior to loading were determined using the Bradford assay (Sigma): samples were mixed with 6x Laemmli sample buffer, and equal protein amounts (100 *µ*g) then underwent electrophoresis on a 10% polyacrylamide gel in running buffer (0.3% Tris base, 1.44% glycine, and 0.1% SDS in distilled water). This was followed by transfer of the proteins onto PVDF membranes in transfer buffer (using 0.3% Tris base, 1.44% glycine, and 20% methanol, in distilled water). Membranes were blocked for 1 h with 5% nonfat milk solution in TBS containing 0.1% Tween 20. iNOS expression was assessed using a specific monoclonal antibody (1 : 1000; Santa Cruz, USA). The signal was amplified with HRP-linked anti-mouse secondary antibody (1 : 2000) and visualized by ECL (Western blotting detection reagent; Amersham Biosciences, USA). Densitometric analysis was performed using NIH ImageJ software and normalised to tubulin loading controls in the same sample.

### 2.9. NOS Activity: Nitrate/Nitrite Production Assays

Animals (*n* = 5) were challenged with LPS and treated with Cbls as described above. At 4 h and 24 h after LPS challenge, lung and liver tissue samples were harvested, homogenized, and processed for determination of NOS activity, as measured by nitrate/nitrite end-products of NO. The ultrasensitive, NOS assay used (Oxford Biomedical Research, Oxford, MI, USA: ultrasensitive colorimetric NOS assay: cat no. NB78) employs an NADPH recycling system—NADP^+^, glucose-6-phosphate, glucose-6-phosphate dehydrogenase and the substrate, L-arginine, but not the cofactor, BH_4_,—to ensure that NOS operate linearly for up to 6 hours, as NO-derived nitrate and nitrite accumulate. The assay kit can accurately measure as little as 1 pmol/milliL (~1 milliM) ^•^NO produced in aqueous solution. In these studies, the assay was run for 5 h at 37°C. The enzyme nitrate reductase was used to convert all nitrate to nitrite, then Griess reagent employed to quantify nitrite levels, with the generation of a nitrite standard, as recommended by the supplier. The completed reaction was read at 540 nm in a Microtiter plate reader. Data are expressed as mmol nitrite/*µ*g protein.

### 2.10. Determination of Tumour Necrosis Factor Alpha (TNF-*α*) and Interleukin (IL-6) Levels

After collection, blood was centrifuged and the plasma separated, under low lighting conditions, then stored at −80°C until performance of the analyses. For determination of circulating TNF-*α* and IL-6 levels, using ELISA assays, samples were diluted 1 : 10 in the assay diluent, as specified by the manufacturer (R&D, UK). Absorbance was plotted in a standard curve, and data expressed as the content of TNF-*α* (ng) or IL-6 (pg) per mL of plasma.

### 2.11. Reagents

Unless otherwise stated, all reagents were purchased from Sigma-Aldrich, Poole, UK.

### 2.12. Statistics

Data are shown as a mean ± S.E. of 5 animals per group for the analyses in non-lethal endotoxaemia and, initially, 7–9 per group, then 12 per group, for lethal endotoxaemia survival, series I and II, respectively. Statistical differences were determined by ANOVA, following the Student Newman Keuls test. Chi-square and Kaplan-Meier tests were used for the lethality studies. In all cases, a *P* < 0.05 was taken as significant.

## 3. Results

### 3.1. Cobalamins Do Not Inhibit LPS-Induced NF-*κ*B Activation *In Vitro*

Since Cbl has been shown to prevent NF-*κ*B activation in a non-immune challenge model [17], and activation of NF-*κ*B leads to iNOS induction, we first looked at the effects of the five principally occurring, intracellular Cbls, (CNCbl, HOCbl, GSCbl, and the two mammalian enzyme cofactors for MU and MS, respectively, AdoCbl and MeCbl), on LPS-induced NF-*κ*B activation *in vitro,* using a canonical reporter assay. Although the various incoming forms of Cbl are all reduced or dealkylated soon after cell entry, prior to MS/MU cofactor formation [60], the different incoming forms affect both the rate and ratio of formation of the two known active cofactors, AdoCbl/MeCbl [60–62]. Theoretically, this variability in Cbl cofactor formation may impact on the effects of Cbls in immune challenge with respect to NF-*κ*B activation. Thus, it was important to make this comparison. RAW 264.7 macrophage cells, stably transfected with NF-*κ*B luciferase reporter construct, were preincubated for 1 h with increasing concentrations (1–10–100 *μ*M) of Cbls. Upon LPS stimulation, none of the five Cbls significantly affected or inhibited LPS-driven NF-*κ*B activation at 1 h, with no significant inhibitory effect on NF-*κ*B at a later time point (24 h). CNCbl alone slightly stimulated NF-*κ*B activity, but only at the 1 h time point and when tested at the concentration of 1 *μ*M (Table 3).

In two separate experiments, each performed in triplicate, RAW246.7 cells, stably transfected with NF-*κ*B luciferase reporter construct, were seeded in 96-well plates and preincubated for 1 h with increasing concentrations (1–10–100 *µ*M) of individual Cbls, followed by stimulation with *E. coli* LPS (1 *μ*g). At 1 h, and 24 h, following LPS in the respective experiments, cells were processed for measurement of luciferase activity. Basal values of fluorescence were 2.60 ±  0.38 and 3.77 ± 0.31 for 1 h and 24 h incubation, respectively. Data are expressed as a mean ± SEM of triplicate observations. *P < 0.05 versus LPS alone.

### 3.2. Endogenous Cobalamins Enhance Survival in Acute Endotoxaemia

As there was no observable difference between the effects of alkyl and non-alkyl Cbls on NF-*κ*B *in vitro*, we chose to focus these first investigations *in vivo *principally on HOCbl, as a clinically licensed Cbl form, known to be partially converted on cell entry to the two Cbl cofactors, MeCbl and AdoCbl, for MS and MCM, respectively [63]. Furthermore, at supraphysiological doses of 5 g i.v., HOCbl, as a clinical cyanide antidote, has shown remarkable protection against corollary inflammation (analogous to the inflammation seen in SIRS, sepsis, and septic shock), that goes beyond merely acting as a magnet for CN [8].

We therefore next decided to see if the lethality survival protection also conferred by HOCbl in a sepsis/endotoxaemia mouse model [7] was reproducible in a different strain and in a more acute endotoxaemia model. Some groups of animals were alternatively treated with the relatively novel, intracellular Cbl, glutathionylcobalamin (GSCbl) [61, 64] whose clinical effects are untested in sepsis, or with N-acetyl-cysteinyl-cobalamin (NAC-Cbl), a synthetic cobalamin, used as a non-endogenously occurring, thiol Cbl comparison. To gain some information on potential clinical dosage, all Cbls were tested in two distinct, high dosing regimes, with or without prophylactic pretreatment.

In a severe sepsis protocol (LPS+D-Gal), using C57BL/6 mice, we administered a relatively low dose of Cbls (0.2 mg/kg i.p.), equivalent to a maximal concentration of approximately 1 *µ*M (considering a total blood volume of 2.5 mL in the mouse, this concentration being well within the range tested *in vitro*). Individual Cbls were administered i.p. −1 h prior to LPS+D-Gal and then given in repeated doses at +1, +2, +6, and +22 h after LPS+D-Gal. Alternatively, a high dose Cbl protocol (40 mg/kg i.p.) was administered only twice, at +2 and +22 h after LPS+D-Gal, to assess its potential as a rescue regimen. The urine of all Cbl-treated animals was red, within 1 h of administration, an indicator of rapid, high, systemic Cbl saturation (data not shown).

**Figure 2:**
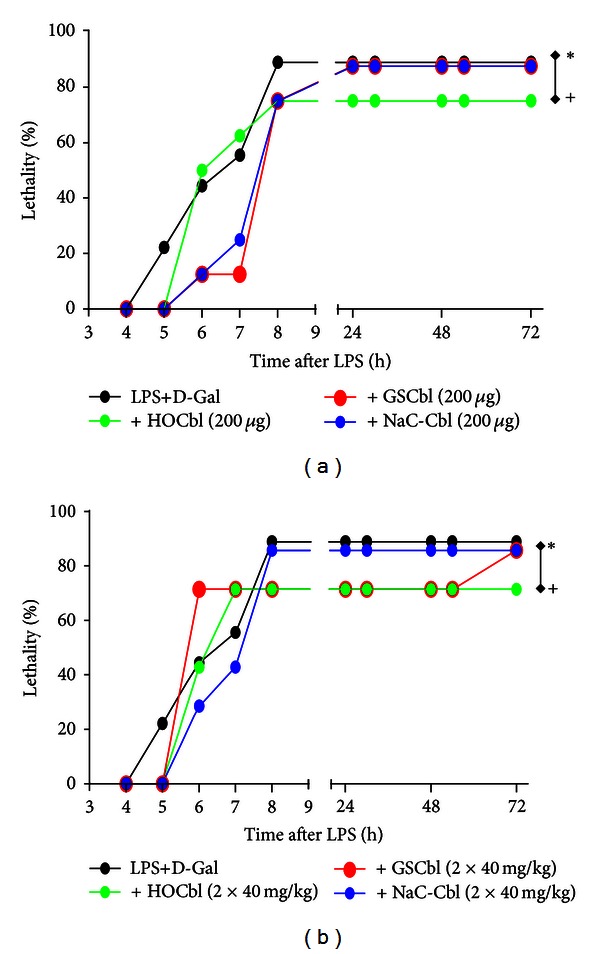
HOCbl protects mice against experimental endotoxaemia. In (a) and (b) mice (*n* = 8 and 7, resp.) were treated with HOCbl or GSCbl or NAC-Cbl following two distinct protocols, in addition to being injected with LPS (0.1 mg/kg i.p.) + D-Galactosamine (1 g/kg i.p.). Impact on lethality was followed for a total of 5 days. (a) Cobalamins were administered at a dose of 0.2 mg/kg i.p. −1 h, +1 h, +2 h, +6 h, and +22 h and compared to controls given LPS+D-Galactosamine injection alone (Time 0 h). *P < 0.05 for GSCbl/NAC-Cbl and + for HOCbl when tested against LPS+D = Gal (*n* = 9), using the Chi-square or Kaplan-Meier tests. (b) Cobalamins were administered at a dose of 40 mg/kg i.p. +2 h and +6 h and compared to controls given LPS + D-Galactosamine injection alone (Time 0 h). *P < 0.01 for NAC-Cbl and + for GSCbl/HOCbl, when tested against LPS+D-Gal (*n* = 9), using the Chi-square or Kaplan-Meier tests.

LPS+D-Gal mice rapidly reached 88.9% mortality by 8 h. This did not change further up to 24 h. Animals treated with the relatively low-dose regimen of GSCbl or NAC-Cbl, were protected in the early, 4–8 h time frame (Figure 2(a)). During this period all Cbl-treated animals also exhibited less huddling and pilo-erection (data not shown).

At 8 h after LPS+D-Gal, all relatively low-dose Cbl treatments afforded 25% survival, ^+^ *P* < 0.05 versus LPS+D-Gal alone (Figure 2(a)). However, only low-dose HOCbl treatment maintained this level of protection up to 24 h (Figure 2(a)). (Indeed, as regards the long-term outcomes, 8 h seemed to be a watershed time point at which the outcome was determined for all groups.) Paradoxically, in view of its early protective effects at the lower dose, the *high-dose* GSCbl regimen was less protective within the first 8 h. High-dose NAC-Cbl, which again provided some degree of protection in the first 6 h, was not significantly different from controls at 24 h. In contrast, high-dose GSCbl and HOCbl, despite the lesser protection of the former in the first hours, offered a consistent 28.60% survival up to 24 h, ^+^ *P* < 0.01 versus LPS+D-Gal alone (Figure 2(b)).

Later, at 72 h following LPS+D-Gal, in the GSCbl high-dose group, mortality was equal to that observed in the NAC-Cbl high dose group, 85.72%, close to that of LPS+D-Gal control animals, though this increase in mortality was a late event: with 28.60% survival to 54 h in this group, perhaps indicative of the general Cbl protective trend. Nonetheless, the 25% and 28.60%, respectively, of mice that were alive at 24 h, in each of the two distinct, low- or high-dose, HOCbl-treated groups, exhibited continued survival up to 72 h, /^+^ *P* < 0.05 versus LPS+D-Gal alone/^+^ *P* < 0.01 versus LPS+D-Gal alone (Figures 2(a) and 2(b)) and beyond (data not shown).

**Figure 3:**
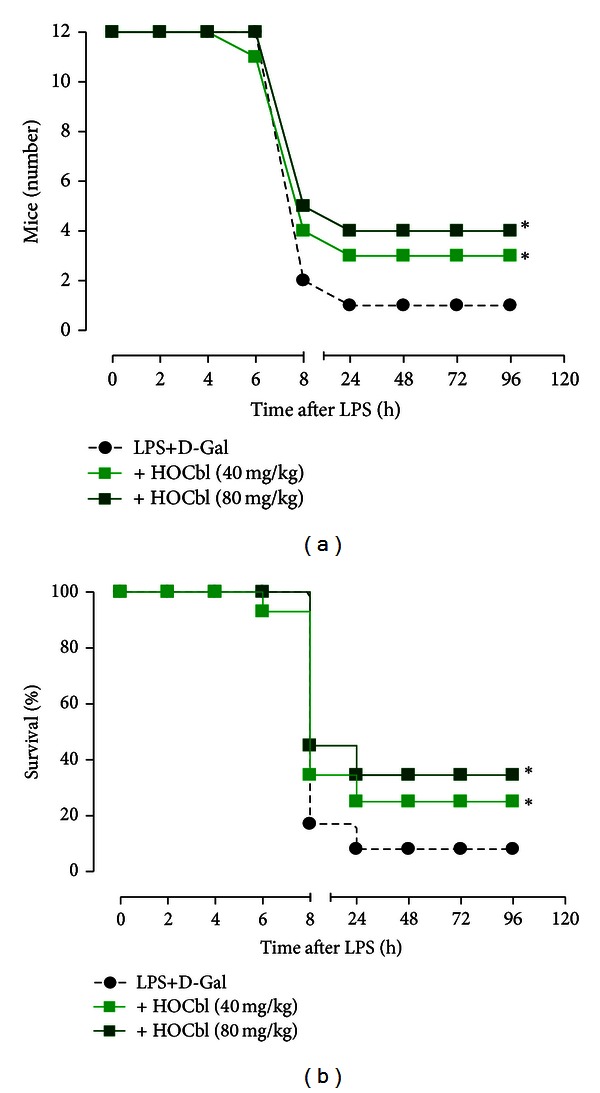
Ultra-high dose HOCbl consistently improves survival in experimental endotoxaemia. Mice (*n* = 12 per group: 2 Cbl active treatment groups plus 1 LPS-only group) were treated with HOCbl, following two distinct protocols, +40 mg/kg at 2 h and +4 h post LPS+D-Gal, and +80 mg/kg at only +2 h following LPS (0.1 mg/kg i.p.) + D-Galactosamine (1 g/kg i.p.). Impact on lethality was followed for a total of 5 days. *P < 0.05 for HOCbl at both doses, when tested against LPS+D-Gal (*n* = 12), using the Chi-square test. *P < 0.01 for HOCbl (80 mg/kg) by using the Kaplan-Meier test.

**II.** Since these initial endotoxaemia studies might be considered underpowered, we repeated the lethality survival experiments using larger groups of mice (*n* = 12) and focussing on HOCbl alone, as having previously shown the most consistent protective effects. This time, given the trend towards improved survival seen at the higher HOCbl dose, two distinct ultra-high doses of HOCbl (40 mg/kg and 80 mg/kg) were tested, with a more concise dose/time frame, +2 h and +4 h only for the 40 mg/kg, and, in the case of the 80 mg/kg dose, a single bolus administration at +2 h. The significant survival advantage of HOCbl treatment results demonstrated over 5 days, in Figures 3(a) and 3(b), not only shows that our HOCbl data is consistently reproducible, but also that increasing the dose of HOCbl significantly increases survival, from 25% up to 33.333%: ^+^ *P* < 0.01 for HOCbl (80 mg/kg) by using the Kaplan-Meyer test. By comparison, at 24 h in the LPS-only group, there was 90% mortality.

To gain information about the mechanisms behind the consistent protection afforded by HOCbl, and to observe any potential impact on the NOS, a non-lethal protocol was next deployed. Then the expression of inflammatory mediator genes in liver and lung was analysed, in both the pro- and anti-inflammatory phases of the immune response, at the 4 h and 24 h time points.

### 3.3. HOCbl Selective Promotion/Modulation of eNOS/iNOS mRNA, Inhibition of IL-1*β*, and Cox-2 Expression: 4 h Time Point

**Figure 4:**
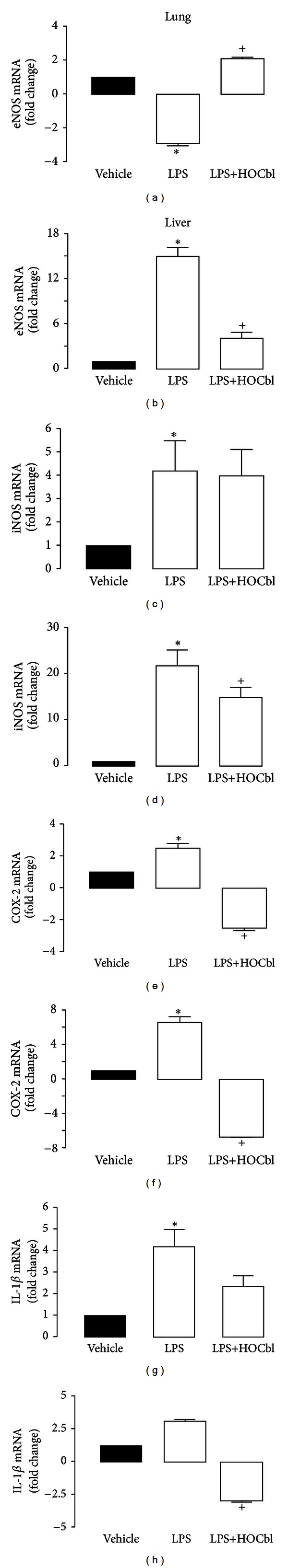
HOCbl selectively modulates NOS enzymes and inhibits COX-2, IL-1*β* mRNA, in lung and liver, at 4 h following LPS-induced endotoxaemia. Mice (*n* = 5) were treated with HOCbl, at the low dose protocol (0.2 mg/kg i.p.) and compared to LPS only (0.1 mg/kg i.p. at time 0 h). Organs (lung and liver) were harvested at 4 h after LPS and gene expression was quantified in tissue extracts by real-time PCR, using GAPDH and RPL32 as internal standards. ((a), (b)) eNOS mRNA data; ((c), (d)) iNOS mRNA data; ((e), (f)) COX-2 mRNA data; ((g), (h)) IL-1*β* mRNA data. Values are a mean ± SEM of triplicate observations. *P < 0.05 versus vehicle (PBS-treated) control; ^+^ *P* < 0.05 versus LPS-only group.

The early effects of HOCbl treatment on eNOS mRNA appeared organ dependent, with significant promotion of eNOS mRNA in the lung and attenuation in the liver (Figures 4(a) and 4(b)). For eNOS, in LPS-only animals we observed a decrease in the lung of −2.9 ± 0.1, whereas there was an increase of 2.1 ± 0.1-fold change in LPS+HOCbl-treated animals (Figure 4(a)). Paradoxically, in the liver of LPS-only treated animals, there was an increase of eNOS expression of up to ~15-fold compared to up to ~4-fold change only in LPS+HOCbl—treated animals (Figure 4(b)).

Liver and lung iNOS and COX-2 gene expression levels were increased in LPS-only treated animals when compared to that of PBS-only injected mice, whose value was set as 1. However, as for eNOS, the effects of HOCbl on iNOS expression were once more organ selective, failing to inhibit the rise in iNOS mRNA in the lung, but attenuating it in the liver (Figures 4(c) and 4(d)). Strikingly, in spite of HOCbl's failure to completely inhibit iNOS expression, HOCbl was a consistent inhibitor of COX-2 mRNA in both liver and lung, bringing its degree of expression back to and below that of PBS-injected mice (Figures 4(e) and 4(f)). HOCbl treatment also had a consistent regulatory effect on IL-1*β* expression, which was moderately and significantly decreased in lung and completely inhibited in liver (Figures 4(g) and 4(h)).

### 3.4. HOCbl Has Early Promotional Effects on Translation of eNOS/iNOS Protein

**Figure 5:**
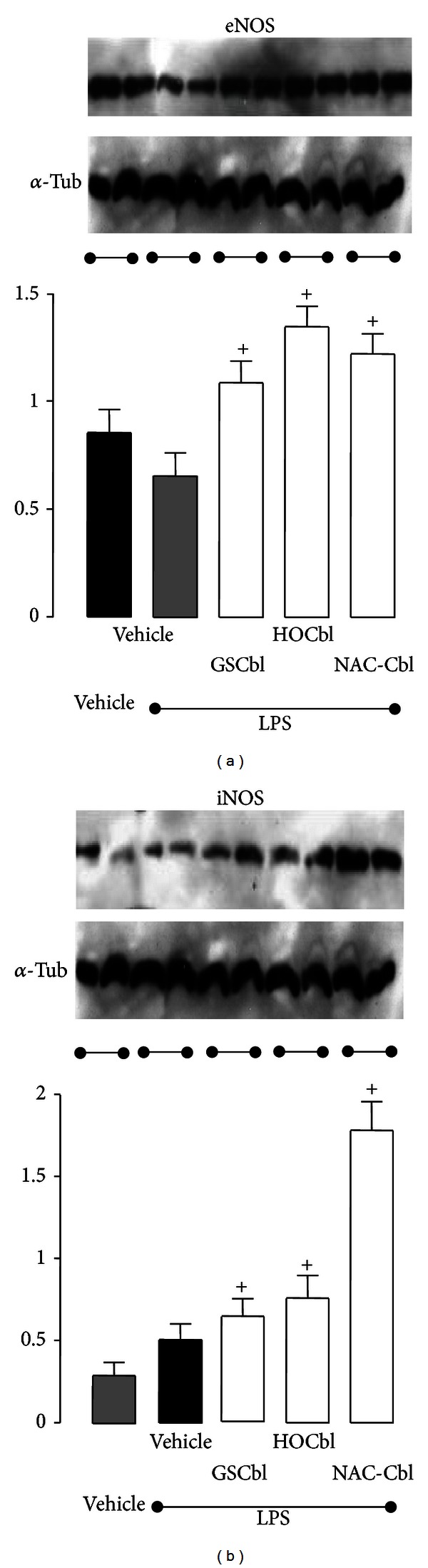
Early (4 h) promotional effect of HOCbl on iNOS and eNOS protein expression in liver samples from endotoxemic mice: mice (*n* = 5) were treated with HOCbl at the low dose protocol (0.2 mg/kg i.p.) at –1 h, +1 h, and +2 h and challenged with LPS (0.1 mg/kg i.p.) at time 0. Liver samples were collected and processed as described in Materials and Methods, for the Western blot analysis of iNOS and eNOS protein expression. Upper panels ((a), (b)) show membranes probed for iNOS, eNOS, with tubulin as the loading control; lower panels show densitometric analysis of the blots. Values are a mean ± SEM of triplicate experiments. *P < 0.05 versus vehicle (PBS-treated) control; ^+^ *P* < 0.05 versus LPS group.

To determine efficiency of translation of the post-LPS increased NOS mRNA, we assessed eNOS and iNOS protein expression by Western blot in 4 h liver samples. As predicted by our hypothesis that sepsis may involve a failure in translation of the NOS, this revealed that whilst in the LPS-only challenged group there was a significant depression of eNOS protein translation, that was at odds with its high mRNA expression, HOCbl significantly promoted eNOS protein translation, above the levels of both PBS control and LPS-only treatment groups (Figure 5(a)). A similar paradoxical pattern was observed in hepatic iNOS protein translation, with significant depression of iNOS protein translation in the LPS-only challenged group, and promotion of iNOS protein in the HOCbl+LPS treated group (Figure 5(b)). We confirmed that these effects of HOCbl on NOS protein promotion were not random or artifactual, but were specific to Cbl, by repeating the LPS non-lethal endotoxaemia experiment using either GSCbl or NAC-Cbl treatment and performing Western blots for eNOS/iNOS protein. Once again, we observed a significant early promotion of eNOS/iNOS protein by these other Cbls, when compared to LPS only (Figures 5(a) and 5(b)).

### 3.5. HOCbl Moderates High ^•^ *NO* Synthesis at 4 h and 24 h after LPS

**Figure 6:**
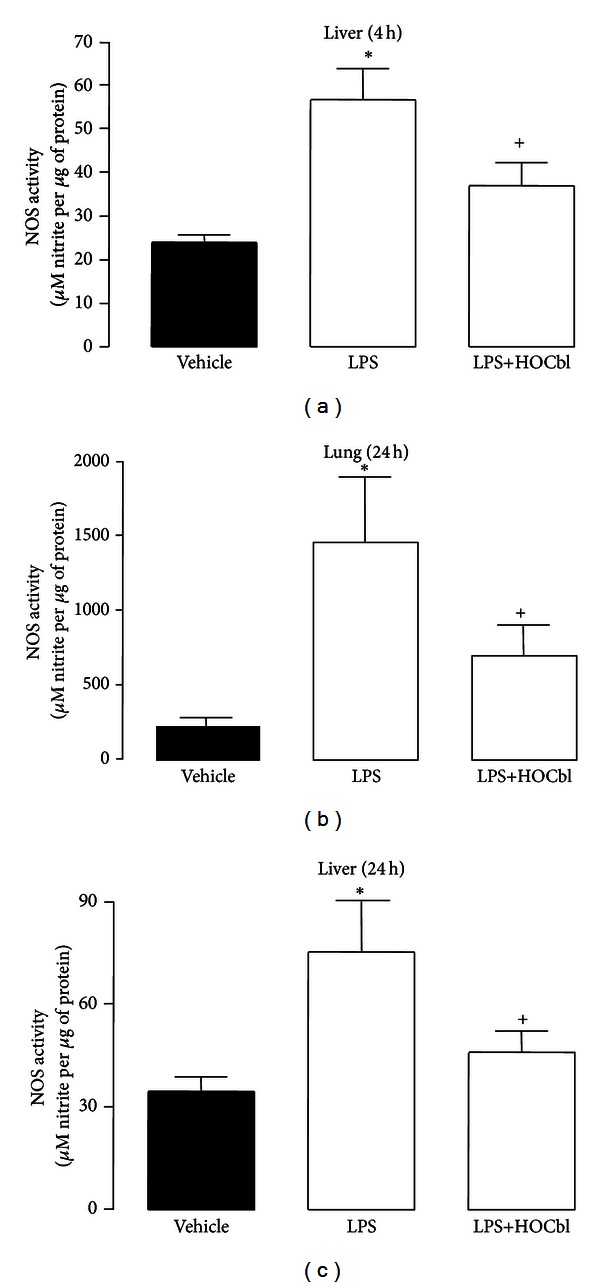
Modulatory effects of HOCbl on NOS activity in tissue homogenates of endotoxemic mice at 4 h and 24 h. Liver ((a), (c)) and lung (b) tissue samples were collected 4 h and 24 h after LPS challenge (0.1 mg/kg; i.p.); two groups of mice (*n* = 5 per group) having previously been treated with HOCbl (resp., 0.2 mg/kg i.p. at −1 h, +1 h, and +2 h and at −1 h, +1 h, +2 h, +6 h, and +22 h) and also challenged with LPS at time 0. NOS activity at 4 h and 24 h was assayed as described in Materials and Methods, and shown as *µ*mol of NO generated per *µ*g protein. Values are a mean ± SEM of triplicate observations. *P < 0.05 versus vehicle (PBS-treated) control; ^+^ *P* < 0.05 versus LPS group.

**Figure 7:**
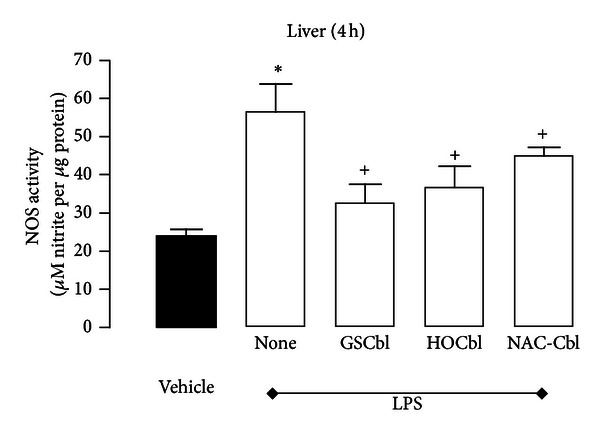
The effects of thiol Cbls on activity of NOS. Liver tissue samples were collected 4 h after LPS challenge (0.1 mg/kg; i.p.); 4 groups of mice (*n* = 5 per group) having previously been treated with either LPS-only or HOCbl/or GSCbl or NAC-Cbl plus LPS at time 0 h (0.2 mg/kg i.p. at −1 h, +1 h, and +2 h and at −1 h, +1 h, +2 h, +6 h, and +22 h). NOS activity at 4 h was assayed as described in Materials and Methods, and shown as *µ*mol of NO generated per *µ*g protein. Values are a mean ± SEM of triplicate observations. *P < 0.05 versus vehicle (PBS-treated) control; ^+^ *P* < 0.05 versus LPS group.

However, when NOS activity was measured (using a nitrite production assay) both in the early post-LPS challenge, pro-inflammatory phase and in the late anti-inflammatory, resolution phase, a further paradoxical result emerged, suggesting that HOCbl may exert some post-translational modification of NOS activity. Levels of nitrite at 4 h showed an inverse relation to levels of NOS protein, with significantly higher levels of nitrite in the LPS-only eNOS/iNOS-depressed group and significantly lower levels of nitrite being generated in the HOCbl/eNOS/iNOS-promoted group (Figure 6(a)). (That this was a general, reproducible Cbl effect was confirmed, as stated previously, by also doing the Western blot with 4 h GSCbl/NAC-Cbl-treated liver samples and also running the NOS activity assay with both thiol-Cbl treated samples.) Here we again observed a correlation between GSCbl/NAC-Cbl promoted high NOS protein in the Western blots and decreased nitrite in the NOS activity assay (Figure 7).

At 24 h following LPS, levels of NOS-derived nitrite, as measured in tissue samples, were even higher than at 4 h in both the LPS-only and HOCbl-treated groups. Nevertheless, HOCbl consistently showed *relatively* less nitrite production than LPS only, in both lung and liver tissue samples (Figures 6(b) and 6(c)).

### 3.6. HOCbl Regulates TNF-*α* Expression and Protein but Leaves Circulating IL-6 Protein Levels Unchanged

**Figure 8:**
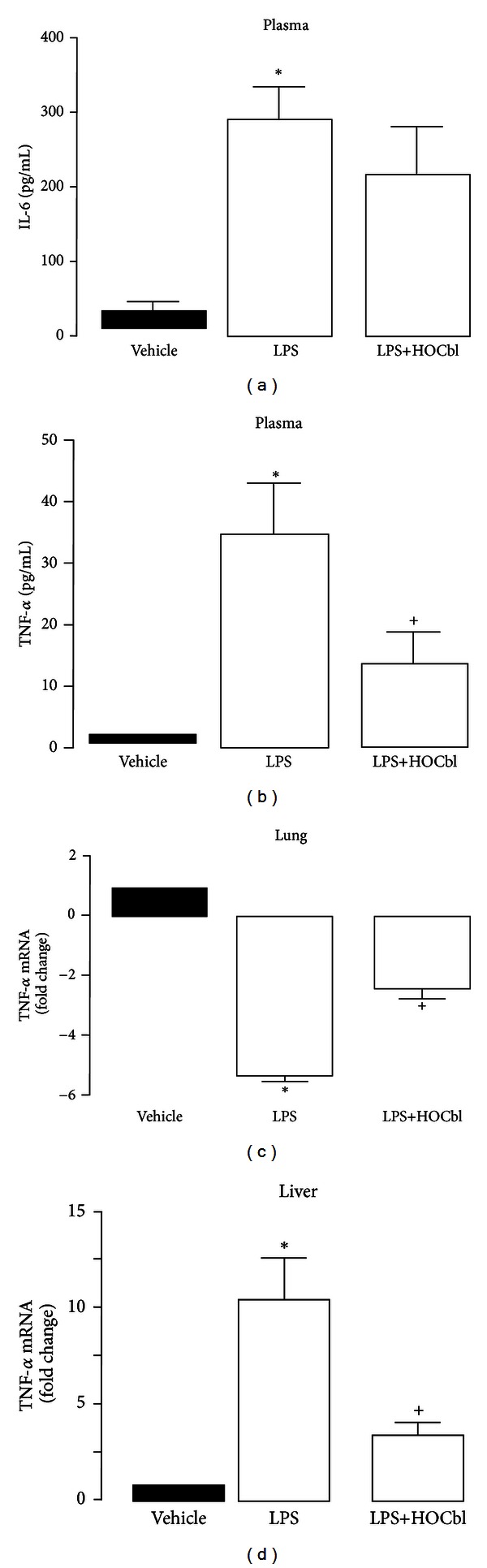
Selective effects of HOCbl on IL-6 and TNF-alpha at 4 h following LPS-induced endotoxaemia. Mice (*n* = 5) were treated with HOCbl, at the low dose protocol (0.2 mg/kg i.p.) at −1 h, +1 h, and +2 h and compared to LPS (0.1 mg/kg i.p. at time 0). Organs (lung and liver) were harvested at 4 h subsequent to LPS, and gene expression quantified in tissue extracts by real-time PCR, using GAPDH and RPL32 as internal standards. ELISA was used to measure plasma levels of IL-6 (a) and TNF-*α* (b). Tissue expression of TNF-*α* mRNA in lung (c) and liver (d) extracts. Data are a mean ± SEM of 5 mice per group. *P < 0.05 versus vehicle (PBS-treated) control; ^+^ *P* < 0.05 versus LPS group.

Since high iNOS expression/protein and ^•^NO activity in the sepsis literature are associated with high TNF-*α*, IL-6, and ensuing toxicity, we evaluated how HOCbl might impact upon systemic levels of TNF-*α* and IL-6 triggered by LPS. Plasma levels of these cytokines were quantified using ELISA. Levels of IL-6 protein were not significantly lower in the HOCbl-treated group (Figure 8(a)) However, HOCbl treatment significantly (*P* < 0.05) attenuated the post-LPS-induced increase in circulating plasma TNF-*α*, as measured at the 4 h time point, (~50% reduction: Figure 8(b)). Consistent with its effects on plasma TNF-*α*, HOCbl also showed some protection from the inhibitory effects of LPS on TNF-*α* mRNA in the lung and significant attenuation of TNF-*α* mRNA in liver (Figures 8(c) and 8(d)).

### 3.7. HOCbl Late Effects on NOS and Cox-2 mRNA: 24 h Time Point

**Figure 9:**
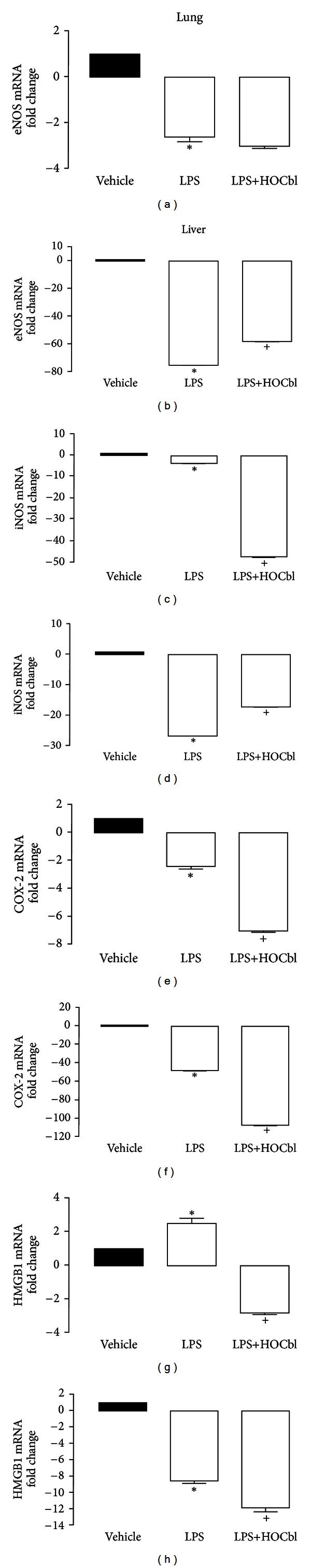
HOCbl modulates eNOS/iNOS, COX-2, HMGB1 gene expression in lung and liver at 24 h subsequent to LPS-induced endotoxaemia. Mice (*n* = 5) were treated with HOCbl (0.2 mg/kg i.p low dose regimen (Table 1)) and compared to LPS 0.1 mg/kg (given i.p. at time 0 h). Organs (lung and liver) were harvested at 24 h after LPS and gene expression was quantified in tissue extracts by real-time PCR, using GAPDH and RPL32 as internal standards, and PBS-injected group set as 1. ((a), (b)) eNOS mRNA data; ((c), (d)) iNOS mRNA data; ((e), (f)) COX-2 mRNA data; ((g), (h)) HMGB1 mRNA data. Values are a mean ± SEM of triplicate observations. *P < 0.05 versus vehicle (PBS-treated) control; ^+^ *P* < 0.05 versus LPS group.

In the resolution phase of the immune response to LPS, the effects of HOCbl treatment on NOS expression displayed a degree of organ selectivity, though most notable with respect to iNOS expression. Whilst HOCbl did not change LPS-induced eNOS mRNA inhibition in the lung, it significantly attenuated its inhibition in the liver, from −75- to −58 fold-change, respectively, for LPS only and LPS+HOCbl (Figures 9(a) and 9(b)).

HOCbl effects on iNOS mRNA in the lung were more distinctive, with ~80% inhibition compared to LPS-only (vehicle group). In the liver, HOCbl treatment attenuated the LPS-induced inhibition of iNOS mRNA by ~40% (Figures 9(c) and 9(d)). The consistent HOCbl tissue inhibition of Cox-2 mRNA, seen at the early pro-inflammatory phase time point of 4 h, persisted at 24 h, showing a significantly greater degree of inhibition than LPS-only: 7- versus 2.5-fold for LPS-only in the lung; 115- versus 50-fold for LPS-only in liver (Figures 9(e) and 9(f)).

### 3.8. HOCbl Inhibition of HMGB1 mRNA at 24 h following LPS

Given the early regulatory impact of HOCbl on NOS/^•^NO activity, and COX-2, IL-1*β*, and TNF-*α*, we expected to see related downstream beneficial effects on expression of the late (≥18 h) effector of endotoxaemia, high mobility group box 1 (HMGB1). This prediction was confirmed. Where LPS-only presented an inconsistent picture, HOCbl treatment consistently inhibited HMGB1 mRNA: in the lung, from an increase of 2.5 in LPS-only to a near threefold decrease (setting levels of expression even lower than those observed in the control group, taken as a value of 1); and in the liver, to a more significant degree, even beyond the remarkable inhibitory effect of LPS-only (Figures 9(g) and 9(h)).

To conclude the 24 h gene expression analyses, tissue levels of IL-1*β* and TNF-*α* mRNA were also quantified using RT-PCR. In resolution, HOCbl treatment significantly increased inhibition of hepatic IL-1*β* (in line with its inhibitory effect at 4 h) and inhibition of TNF-*α* in the lung, whilst also, paradoxically, decreasing LPS inhibition of TNF-*α* in the liver (Table 4). Of note was the fact that the late effects of HOCbl on TNF-*α* mirrored the degree of late iNOS expression, in both lung and liver, as, indeed, did LPS-only (Figures 9(c) and 9(d) and Table 4).

## 4. Discussion

Our studies present a picture of complex and far-reaching homeostatic regulation of the activation, expression, and translation of NOS, ^•^NO synthesis, and inflammatory mediators by HOCbl during the immune response. We propose that this regulation accounts for the noted survivals in rodent endotoxaemia, both in our more acute, septic shock models I and II (a modest but significant 25%/28.60%, and 25%/33.333%, survival) and in a previous sub-acute, sepsis model (performed with CNCbl/HOCbl—*n* = 10 animals per group—30%/40% survival [7]). The regulation we show here may also explain the observed organ/tissue-protective effects of HOCbl in the clinical treatment of CN poisoning and the ensuing shock, which appear to go beyond what may be expected from the Cbl binding of CN alone [8]. Furthermore, given the supraphysiological, saturating doses of Cbl used in our studies, if Cbl had been acting just as an ^•^NO scavenger, or even as a NOS inhibitor, it seems unlikely that it would have permitted the increasing rise in NOS activity (as indexed by nitrite), both early and late, or that its effects would be so subtle, complicated, and ultimately beneficial.

### 4.1. Paradoxes of Thiol Cobalamin Effects

The thiol Cbls, on the other hand, present some mysteries, about which one can only give speculative answers at this point. The survival advantage exhibited in the early hours by comparatively low dose GSCbl/NAC-Cbl treated animals, but not with HOCbl treatment, may be due to the known higher reduction potential/lability of thiol Cbls [64], resulting in more rapidly available intracellular Cbl. Cbl is likely to be, at least in part, functionally deficient in sepsis, as indexed by the down-regulation of the Cbl receptors, megalin and cubilin, in the kidneys of endotoxaemic mice [65], the kidney being known, as a Cbl homeostatic regulator, to reduce its Cbl uptake in states of Cbl deficiency [66]. Pertinently, megalin is down-regulated via the LPS-induced ERK1/2 signaling pathway [67], through which, as noted earlier, both Cbl and ^•^NO achieve their coincidentally regulatory, beneficial effects.

Although the beneficial supply of some extra GSH, as a consequence of thiol Cbl lability, might be proposed as an alternative explanation for early survival protection, it is noteworthy that, at the higher dose, GSCbl showed no early hours protection. Moreover, in a new series of endotoxaemia lethality, *in vivo* survival studies that compared protective effects of *all* endogenous Cbls against those of GSCbl and NAC-Cbl, we observed that though GSCbl/NAC-Cbl still consistently conferred the same early/pre-8-hour protection compared to the 4 other Cbls, paradoxically, both thiol Cbls repeatedly produced poor long-term survival outcomes, equivalent to or worse than LPS-only, whereas HOCbl/CNCbl/MeCbl/AdoCbl all consistently showed significant protection, with CNCbl/MeCbl in particular, producing even better survival results than the current HOCbl-centred study (Brancaleone, Dalli et al. 2011, unpublished data).

GSCbl is certainly known to produce a more rapid early increase in MS activity (together with fourfold greater formation of AdoCbl), when compared to HOCbl [61]. Such an increase in MS activity, normally rapidly deactivated by oxidative stress [68–70], would increase synthesis of the methyl donor, S-adenosylmethionine (SAM), which inhibits LPS-induced gene expression by modulating histone methylation [71]. Whilst this may have initial short-term benefits, as observed, there are also negative long-term consequences from excessive inhibition of the necessary, pro-inflammatory gene expression at this stage. This may be why MS expression and activity are *transiently* decreased by 25% and 30% *early on* in the normal pro-inflammatory phase of the immune response to LPS [72], as well as to allow for GSH synthesis modulation [72].

The consequent, equally paradoxical failure of the other thiol Cbl, NAC-Cbl, to significantly increase survival beyond 8 h, at both high- and low-dose protocols, may also be attributable to the fact that NAC is particularly unstable as a Cbl ligand and may therefore have acted independently of Cbl as NAC alone. Further, though NAC can (1) act as an antioxidant by increasing GSH levels (2) it can equally act as a pro-oxidant [73], increasing disulfides, GSSG, [74] and (3) is counter-indicated in sepsis, since, whilst NAC enhances phagocytosis, it also suppresses the bactericidal respiratory burst in ICU patients, with potential negative outcomes [75].

Indeed, consistent with this independent, paradoxical observation, additional data from our *in vivo* endotoxaemia model shows that in the early 4 h phase of the immune response NAC-Cbl, in contrast to HOCbl/GSCbl, significantly increases circulating PMN, specifically granulocytes, yet decreases the intensity of CD11b expression (see Supplementary data available online at http://dx.doi.org/10.1155/2013/741804 (and Researchgate)). The adhesion molecule/complement receptor, CD11b, is a marker of neutrophil activation, and its high expression normally correlates with a strong respiratory burst [76].

It is conceivable then that such NAC-promoted suppressive effects ultimately outweighed the very early benefits in survival protection conferred by NAC-Cbl.

### 4.2. Cbl, NF-*κ*B, and iNOS

The initial *in vitro* observation that all major endogenous Cbls do not inhibit NF-*κ*B activation, even at 24 h, may appear surprising, particularly in view of the Cbl beneficial outcomes *in vivo.* However, it has previously been shown *in vivo* that early inhibition of NF-*κ*B in immune challenge increases and prolongs inflammation, and that persisting late NF-*κ*B activation (24/48 h) permits its resolution [77]. Moreover, this failure to inhibit NF-*κ*B by Cbl during inflammation, with positive outcomes, has now also been observed by a group who were aware of our findings [57] and, independently, in a Cbl cancer model by Marguerite et al. (2012, Marc Marten personal communication to Wheatley). 

**Figure 10:**
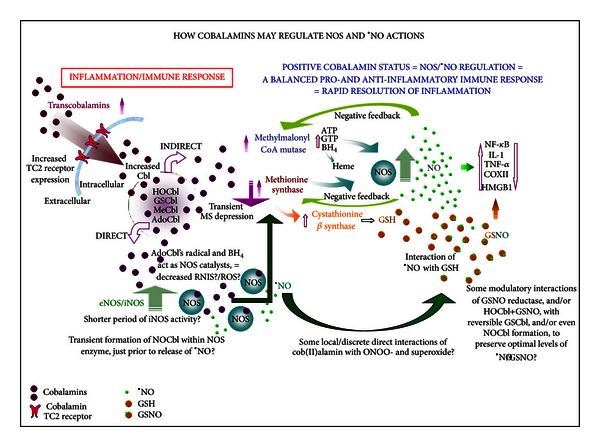
Hypothetical scheme showing how cobalamins may modulate the NOS/^•^NO species, both indirectly and directly, with downstream effects, during the course of the immune response. Immune challenge upregulates—via the AP-1/Ras signalling pathway—the circulating Cbl carrier proteins, haptocorrins, /HC-TCI & III (which, when desialylated, are taken up by the hepatic Ashwell receptor, thus transporting more MeCbl to the liver for increased synthesis of acute phase proteins). At the same time, the Cbl tissue carrier proteins, the transcobalamins, /TCII are also upregulated, as a result of crosstalk between the transcription factors, NF-*κ*B and Sp-1, the TCII promoter. The increased supply of intracellular Cbl is partly converted to the active cofactors, AdoCbl and MeCbl, in a high AdoCbl to low MeCbl ratio. When methionine synthase/MS, along with SAM synthesis, is initially decreased by LPS challenge in activated monocytes/macrophages, methylmalonyl CoA mutase/MU activity takes precedence [91], triggering increased expression of TCII cell receptors. Part of the consequent extra AdoCbl synthesised may then be directed towards NOS catalysis. A combined proportional increase in activity of MU and, later, MS ensures that all the necessary components for NOS synthesis/assembly, substrates and cofactors, are in plentiful supply. This prevents excess generation of ROS and RNIS during NOS catalysis. These are further decreased because part of the ^•^NO produced may combine with GSH, or other thiols, to form the more beneficial, antioxidant *S-*nitrosothiols/*S*-NOS. ^•^NO is then released, as needed, in targeted amounts, by the action of GSH-dependent GSNO reductase and/or possibly by interaction with HOCbl, yielding GSCbl and ^•^NO. Increasing levels of GSNO/^•^NO rapidly downregulate IL-1*β*, COX-2, TNF-*α*, HMGB1 expression, and, at the conclusion of the second, anti-inflammatory phase of the immune response also inhibit iNOS, NF-*κ*B, and down-regulate MU, MS, and CBS. The lower right part of the scheme shows a possible direct catalytic regulation of iNOS by AdoCbl's lower axial ligand base, the dimethylbenzimidazole (DMBI), and the adenosyl radical, generated after NOS enzyme-induced homolysis of the Co-C bond, which would reduce formation of RNIS/ROS, increase the ratio of ^•^NO/GSNO to ONOO- and related species, and make NOS catalysis more productive, thereby lessening the duration of iNOS activity and ensuring the beneficial effects of ^•^NO predominate (see [43]).

Further, since activation of NF-*κ*B is linked to induction of iNOS, and since inadequately low levels of ^•^NO—and, indeed, iNOS gene knockout in mice [78]—and NOS inhibitors in clinical trials [79, 80] have been implicated in sepsis morbidity and mortality [81], we adopted the hypothesis that NOS translation may, in contradiction to the common view, actually be depressed in sepsis and NOS catalytic activity ‘‘uncoupled” or malfunctioning. Previously observed high ^•^NO in sepsis is believed to comprise a greater ratio of the more toxic ^•^NO species, such as peroxynitrite, ONOO- [82], which inhibits eNOS [83], as opposed to more antioxidant/cytotoxic ^•^NO forms, such as *S*-nitrosothiols/GSNO [84, 85] or ^•^NO itself; although overhigh levels of GSNO also have negative effects in sepsis-like inflammation [85]. Since Cbl is known to promote GSH [3, 86–88], whose synthesis is induced simultaneously with that of iNOS [89], it should theoretically alter the ratio of GSNO/^•^NO to ONOO- and related species [43], so that the more positive actions of ^•^NO predominate [42] (Figure 10). Therefore, we predicted that Cbl would not inhibit iNOS expression and translation early on. This proved correct, with significant HOCbl (and GSCbl/NAC-Cbl) early promotion of both iNOS and eNOS proteins and significantly lower LPS-only iNOS/eNOS protein. Since eNOS is known to be depressed in sepsis, with adverse cardiovascular consequences [90], this early effect of Cbl may have positive clinical implications.

There is an apparent contradiction in the data showing relatively low iNOS/eNOS mRNA leading to strikingly high protein translation in the LPS+HOCbl treated animals, in direct contrast to the LPS-only group, where strikingly high iNOS/eNOS mRNA yielded much lower levels of iNOS/eNOS protein. That this is not an artefact, but a specific Cbl effect, also observed by others [47], is seen by the comparable inverse results achieved with the two, thiol Cbls. (These collective Cbl results were also mirrored by decreasing nitrite/NOS activity, in inverse proportion to the ascending levels of HOCbl/GSCbl/NAC-Cbl iNOS protein.)

We propose that this paradox may actually be an index of Cbl/NOS regulation in endotoxaemia and that the high mRNA levels in LPS-only animals may be due to the observed phenomenon of “relaxed control of RNA synthesis” when SAM/methyl groups are deficient [92], as in folate or Cbl deficiency [93], thus consequential on the functional Cbl deficiency of endotoxaemia/sepsis, and more permanent MS inactivation by LPS, discussed earlier. Furthermore, it is theoretically possible that abnormal cell function in sepsis may result in much of the mRNA produced by the LPS-only group being “masked” and unavailable for efficient translation. It is possible also that the translated protein may be unstable and degrade at a faster rate. This is certainly known to be the case with eNOS mRNA in hypoxia [94] and in the presence of high TNF-*α* [95], both characteristic of sepsis. In contrast, given Cbl's impact on the two coenzymes, MS and MU, upon Cbl treatment, the septic cell should afford a degree of metabolic normality and thus economic efficiency in transcription/translation.

A remarkable study, over half a century ago, demonstrated that Cbl is capable of reactivating a diversity of key enzymes after acute oxidant stress, most of them also negatively affected in sepsis, including glucose-6-phosphate-dehydrogenase, lactate dehydrogenase, lysine, ornithine, and glutamic decarboxylase [96]. This last is critical for the supply of alpha-ketoglutarate in the Krebs cycle, which is depressed in sepsis, with consequent lower ATP production that is associated clinically with increased mortality [97]. In turn, supply of alpha-ketoglutarate determines the availability of L-arginine, glutamate, and glutamine, all of which also decrease in sepsis, with adverse consequences, in the case of L-arginine especially for the NOS [98]. Observed low levels of L-arginine in sepsis [99] are associated with increased reactive nitrogen species, including high ONOO-, and reactive oxygen species production during NOS catalysis [98], interfering with the normal, more beneficial ^•^NO cell signalling, necessary for the efficient resolution of the pro-inflammatory phase of the immune response.

### 4.3. HOCbl Reciprocal Regulation of iNOS/NO and TNF-*α*

iNOS-derived ^•^NO is known to have a direct regulatory correlation to levels of TNF-*α* [100, 101]. This iNOS/^•^NO/TNF-*α* regulation seems operative here with HOCbl treatment, not LPS-only. Since HOCbl permitted a moderate rise in ^•^NO (at least, as measured by NOS nitrite end-products), in tandem with moderate levels of TNF-*α* mRNA and protein, and since Cbl status has an inverse relation to TNF-*α* levels [15], it seems reasonable to conclude that such Cbl/TNF-*α* regulation occurs downstream of HOCbl/NOS/^•^NO regulation. Additional evidence for this in our studies may be seen even in the resolution phase of the immune response, where HOCbl-related levels of iNOS expression showed a direct correlation to those of TNF-*α*, even to the degree of inhibition, in both lung and liver tissues (Figures 9(c) and 9(d)/Table 3). Importantly, however, in the early pro-inflammatory phase HOCbl iNOS/^•^NO regulation does not completely inhibit TNF-*α*: 50% reduction being observed in our experiments. This is a critical point as anti-TNF-*α* mAb treatment increases sepsis mortality in the clinic, since some degree of TNF-*α* production and consequent early pro-inflammatory signalling is essential for an effective immune response [102].

### 4.4. HOCbl Regulation of IL-6, IL-1*β*, and Cox-2

Also noteworthy, and crucially consistent with such HOCbl/iNOS/TNF-*α* regulation, is HOCbl's apparently selective failure to inhibit IL-6 early on. IL-6 is essential for induction of acute phase proteins, whilst simultaneously also decreasing pro-inflammatory cytokines and increasing anti-inflammatory factors [103]. IL-6 regulation of pro-inflammatory factors includes regulation of TNF-*α* and IL-1*β* [104], expression of the latter also determining that of Cox-2, involved in arachidonic acid-derived prostaglandin and leukotriene synthesis [105]. IL-1*β* is rapidly expressed (~15 min post LPS) [106], whereas iNOS, which may be induced by IL-1*β*, is not fully expressed until 6 h after LPS [107].

We have observed that treatment with high doses of endogenous Cbls (HOCbl/GSCbl) promotes iNOS mRNA expression as early as 2 h following LPS (unpublished data), possibly fast forwarding the immune response. This, together with the HOCbl-promoted high NOS protein, controlled rise in ^•^NO synthesis, and thence a moderate production of TNF-*α*/IL-6 may form a feedback loop accounting for the tight HOCbl regulation of IL-1*β*, and consequently also Cox-2, as seen at 4 h after LPS (Figure 10 scheme).

### 4.5. HOCbl Inhibition of Late HMGB1 Gene Expression

Cobalamin-promoted NOS/^•^NO early regulation of TNF-*α*/IL-6/IL-1*β*/Cox-2 seems also to be consistent with, and accounts for, the later inhibition of HMGB1 mRNA. If expressed at >18 h and then released extracellularly, HMGB1 can trigger further late release of TNF-*α*, IL-1*β*, and inflammatory products from COX-2, iNOS, and excessive ROS and RNI species, leading to pathology [108]. It is known that the nervous system can modulate circulating TNF-*α* levels *via* release of acetylcholine by the vagus nerve [109]. But our studies show that Cbl—essential for acetylcholine synthesis [110]—is the first known endogenous inhibitor of late HMGB1 mRNA expression. Both nicotine and ethyl-pyruvate have been used to block extracellular release of HMGB1 [109] but, in addition to the fact that HOCbl appears to impact on HMGB1 much further upstream, at least in tissues, neither drug is endowed with the safety profile of Cbl [111] and, more pertinently, neither is known to exert such a central, endogenous regulation of the immune response. Further Cbl/sepsis studies should include measurement of plasma HMGB1 levels. But, on the evidence of the general anti-inflammatory regulation observed in our studies, we predict that extracellular release of HMGB1, from macrophages and PMN, should be negligible with HOCbl treatment.

### 4.6. A New Paradigm for the Cbl/NOS/^•^NO Relationship?

The theory that Cbl may impact on the NOS indirectly, through the contribution of its two known mammalian coenzymatic functions to NOS substrate and cofactor assembly and, indeed, to assembly of the NOS protein itself [39], may further explain our findings, including the Cbl-promoted high NOS protein (Figure 10 scheme). Furthermore, a deficiency of any of the NOS substrates and cofactors (the likely result of Cbl functional deficiency in endotoxaemia) is known to result in less tightly “coupled” NOS activity and increased free radical generation [112, 113], with a corollary increase in inflammatory mediators and prolonged period of NOS activity, indexed by our observed higher LPS-only NOS nitrite levels.

(In a forthcoming study we will also analyse more exactly how Cbl may shift the ratio of ^•^NO/GSNO/ONOO and related species).

It may also be that Cbl, as AdoCbl and its radical, takes a *direct*, active part in NOS catalysis, as a third mammalian Cbl cofactor [43]. From this perspective, the high NOS protein seen with high Cbl administration may be a classical instance of the cofactor promoting coenzyme assembly. Such a central, direct Cbl/NOS, catalytic interaction would further reduce excess production of toxic forms of ^•^NO, as well as superoxide and other related ROS and RNI species. The consequent, more precise, pro- and anti-inflammatory signalling should again result in a shorter, more effective period of NOS activity, thus lower detectable nitrite levels (as seen) with the beneficial signalling and antioxidant effects of ^•^NO predominant. However, direct Cbl scavenger interactions, in discrete intracellular compartments, with primarily toxic RNIS, such as ONOO-/ONOOH/NO_2_, with which Cbl interacts *ex vivo*, [114], may also play a part and cannot be ruled out as contributing to a more complex picture behind our observed results (Figure 10).

## 5. Conclusions

These novel observations on the mechanism behind cobalamin protection in endotoxaemia suggest that we may be looking at the ideal natural, selective and collective regulator of the NOS, and thence of cytokines and other pivotal factors, in immune challenge and sepsis. In fact, it is now accepted that anti-inflammatory therapies (based on blocking a specific mediator) fail *toutcourt* in sepsis and that a more *modulatory *approach, which regulates the homeostatic inflammatory response, (in itself beneficial), could be successful. Thus, our findings may have significant clinical implications, not only for the treatment of sepsis, but also for other analogous inflammation-driven conditions, such as cancer and malaria, where NOS/^•^NO deregulation, and consequent loss of control over key inflammatory mediators, are equally pathogenic [115, 116].

## Supplementary Material

The Supplementary Data contains material, which casts further light on the immune response effects of treatment with HOCbl, and/or the thiol Cbls, during *in vivo*, experimental endotoxaemia. This material comprises blood analyses of circulating neutrophils/PMN, and of the adhesion molecule/complement receptor, CD11b, a marker of neutrophil activation; hepatic/lung analyses of Vascular Endothelial growth factor gene expression, and also of Asgr1/ or Ashwell receptor, the hepatic receptor for the circulating Cbl carrier, haptocorrin or transcobalamin 1 (HC/ TC1). (Figures: 1-7).
Only some of this material is referred to in the paper. Further reference may be made to it in future publications.

## Figures and Tables

**Table 1:** Protocol for cobalamin treatment of LPS-induced endotoxaemia.

Experimental protocols	Inflammatory stimulus	Treatment	
		Cbl dose	Time of Cbl treatment
4 h non-lethal	LPS (0.1 mg/kg)	0.2 mg/kg	−1 h, +1 h, +2 h
24 h non-lethal	LPS (0.1 mg/kg)	0.2 mg/kg	−1, +1, +2, +6, +22 h
Lethal series I	LPS (0.1 mg/kg)	0.2 mg/kg	−1, +1, +2, +6, +22 h
	+ D-Gal (1 g/kg)	40 mg/kg	+2, +22 h
Lethal series II	LPS (0.1 mg/kg)	40 mg/kg HOCbl	+2, +4 h
	+ D-Gal (1 g/kg)	80 mg/kg HOCbl	+2 h

**Table 2:** 

Assay code	Gene and accession number of detected transcripts
QT00100275	NOS2; NM_010927
QT00152754	NOS3; NM_008713
QT00247786	HMGB1; NM_010439
QT00104006	TNF-*α*; NM_013693
QT01048355	IL-1*β*; NM_008361
QT01658692	GADPH; NM_008084
QT01752387	RPL32; NM_172086

**Table 3:** Effects of the naturally occurring endogenous cobalamins in the NF-*κ*B gene reporter assay.

Treatments	NF-*κ*B reporter assay (fold increase over basal)	
	1 h	24 h
LPS	5.63 ± 0.37	4.60 ± 0.21
CNCbl (*μ*M)		
1	*7.10 ± 0.30	5.70 ± 0.90
10	5.53 ± 0.29	6.60 ± 1.70
100	5.23 ± 0.62	5.53 ± 0.74
GSCbl (*μ*M)		
1	6.33 ± 0.37	5.66 ± 0.67
10	6.25 ± 0.32	5.63 ± 0.63
100	5.98 ± 0.26	5.17 ± 0.84
MeCbl (*μ*M)		
1	6.10 ± 0.10	6.53 ± 1.74
10	6.10 ± 0.15	5.11 ± 1.06
100	6.00 ± 0.58	5.26 ± 0.62
HOCbl (*μ*M)		
1	5.67 ± 0.20	5.73 ± 0.91
10	5.98 ± 0.26	5.57 ± 0.78
100	7.00 ± 1.10	6.10 ± 0.66
AdoCbl (*μ*M)		
1	6.23 ± 0.39	5.36 ± 0.77
10	6.26 ± 0.90	5.40 ± 0.70
100	6.20 ± 0.61	4.53 ± 0.32

**Table 4:** Effects of HOCbl on pro-inflammatory cytokine gene expression, at 24 h following LPS-induced endotoxaemia.

Gene	Organ	LPS	LPS + HOCbl	*P* value (versus LPS)
IL-1*β*	Liver	− 8.30 ± 0.30	−10.6 ± 0.3*	0.0056
	Lung	2.70 ± 0.50	2.1 ± 0.1	0.3046
TNF-*α*	Liver	− 86.90 ± 0.10	−68.6 ± 0.2*	<0.0001
	Lung	− 2.10 ± 0.20	−11.4 ± 0.1*	<0.0001

Mice were treated with HOCbl at the low dose protocol (0.2 mg/kg i.p. at −1 h, +1 h, +2 h, +6 h, and +22 h) and compared to LPS only (0.1 mg/kg i.p. at time 0 h). Twenty-four hours after LPS stimulation, organs (liver and lung) were harvested and processed for assessment of IL-1*β*, TNF-*α*, gene expression by real-time PCR, using GAPDH and RPL32 as internal standards. Values are a mean ± SEM of triplicate observations. *denotes statistical significance for *P* < 0.05.
