# Supplementary material for: Biphasic Modulation of NOS Expression, Protein and Nitrite Products by Hydroxocobalamin Underlies Its Protective Effect in Endotoxemic Shock: Downstream Regulation of COX-2, IL-1β, TNF-α, IL-6, and HMGB1 Expression
Source: Mediators Inflamm. 2013 May 28;2013:741804. doi: 10.1155/2013/741804 (PMC3679756; doi:10.1155/2013/741804)
Supplement: Supplementary file 1 — The Supplementary Data contains material, which casts further light on the immune response effects of treatment with HOCbl, and/or the thiol Cbls, during in vivo, experimental endotoxaemia. This material comprises blood analyses of circulating neutrophils/PMN, and of the adhesion molecule/complement receptor, CD11b, a marker of neutrophil activation; hepatic/lung analyses of Vascular Endothelial growth factor gene expression, and also of Asgr1/ or Ashwell receptor, the hepatic receptor for the circulating Cbl carrier, haptocorrin or transcobalamin 1 (HC/ TC1). (Figures: 1-7). Only some of this material is referred to in the paper. Further reference may be made to it in future publications. [file 741804.f1.docx]

**Biphasic modulation of NOS expression, protein and nitrite products by hydroxocobalamin underlies its protective effect in endotoxaemic shock: downstream regulation of COX-2, IL-1β, TNF-α, IL-6 and HMGB1 expression.**

André LF Sampaio**^1, 2^**, Jesmond Dalli**^1^**, Vincenzo Brancaleone**^1, 3^**, Fulvio D'Acquisto**^1^**, Mauro Perretti**^1+^** and Carmen Wheatley**^4, 5+§^**^,^

**^1^** The William Harvey Institute, Barts and The London School of Medicine and Dentistry, Queen Mary University of London, Charterhouse Square, London EC1M 6BQ, United Kingdom. **^2^** Far Manguinhos – FIOCRUZ, R Sizenando Nabuco, 100, Rio de Janeiro, Brazil CEP21041-250. **^3^** University of Basilicata, Department of Science, Potenza, Italy. **^4^** Orthomolecular Oncology, reg. Charity no: 1078066, 4, Richmond Road, Oxford, OX1 2JJ, United Kingdom. **^5^** St Catherine’s College, Manor Road, OX1 3UJ, Oxford University. ^+^These authors share senior authorship.

**Supplementary data. Blood analyses of PMN, CD11b: hepatic/lung analyses of Vascular Endothelial growth factor, and TC1/Asgr1/Ashwell receptor.**

**(Figures: 1-7).**

**_____________________________________________________________________**

**Effects of cobalamins on blood leukocyte counts during LPS endotoxaemia**

We observed at 4 hours after LPS injection (100 µg/kg) a significant leukopenia due to a decrease in mononuclear cell number, without changes in PMN population. Treatment with the cobalamins (200 µg/kg) was performed 1h before and 2h after LPS challenge. In this condition, HOCbl and GSCbl were unable to affect the response to LPS. On the other hand, animals treated with NAC-Cbl showed increased PMN numbers when compared to LPS- or vehicle-injected animals, without any changes in the total number of leukocytes or mononuclear cells (Figure 1).

**Effects of cobalamins in CD11b expression in blood granulocytes (GR-1^high^ cells) during LPS endotoxaemia**

LPS challenge did not change the PMN numbers as assessed by light microscopy (Fig 1C), this observation was mirrored by the FACS analysis of GR-1^high^ positive cells, a marker for granulocytes (Figure 2A). The numbers of GR-1 ^high^ cells in the cobalamin-treated groups also followed the same trend observed by the light microscopy analysis (Figure 2A).

The level of expression of the integrin CD11b was assessed as a marker of neutrophil activation. The numbers of the double positive population CD11b/GR-1 ^high^ in the blood was not altered after LPS challenge; however differently from what observed for the GR-1^high^ population globally, NAC-Cbl significantly altered the number of CD11b/GR-1 ^high^ double positive cells in the blood (Figure 2B).

Analysis of the intensity of CD11b expression unveiled a different pattern with an increase of CD11b expression after LPS challenge and a significant decrease in the NAC-Cbl, treated group (Figure 2C).

**Assessment of neutrophil accumulation into lung tissue during LPS endotoxaemia**

Lung is a shock organ in the pathogenicity of sepsis and endotoxaemia, herein we analysed the extent neutrophil accumulation in lung tissue 4h after LPS challenge. In these conditions, LPS challenge (100 µg/kg) failed to trigger neutrophil accumulation into lung tissue (Figure 3).

**Effects of cobalamins on Asgr1 gene expression triggered by LPS**

At the 4 h time-point, LPS endotoxaemia triggered a 90-fold increase in Asgr1 gene expression in the liver (Figure 4). Treatment with HOCbland GSCbl significantly reduced this augmented degree of Astgr1 gene expression, however, NAC-Cbl abrogated this response restoring the expression to the value observed in Vehicle-injected animals. In the lungs, on the other hand, the Asgr1 gene expression was downregulated upon LPS treatment, and effect, which is further potentiated upon treatment of the animals with the HOCbl and NaC-Cbl whereas treatment of mice with the GSCbl produced no further change with respect to LPS effect.

**Effects of cobalamins on vascular endothelial growth factor expression post- LPS.**

The analysis of VEGF1 and VEGF2 (Figure 5B & 5C, respectively) unveiled an interesting pattern of expression. VEGF1 gene expression was increased after LPS challenge whereas VEGF2 gene expression was decreased. In this scenario, only the treatment with NAC-Cbl was able to alter this profile decreasing VEGF1 gene expression and restoring VEGF2 gene expression to values comparable to the observed in animals injected with vehicle.

**_____________________________________________________________________**


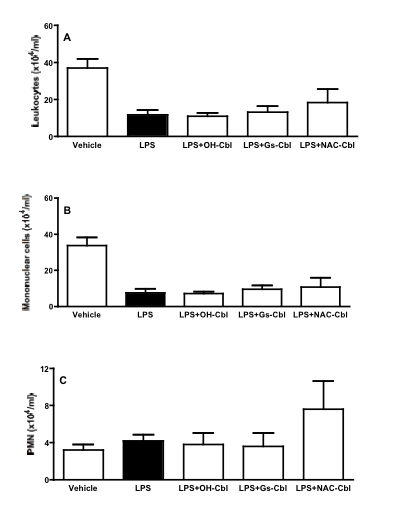


**Figure 1 - Effect of Cobalamins on blood leukocyte counts during experimental endotoxaemia.** Mice were injected i.p. with cobalamins (200μg/kg) 1h prior and 2 h after LPS (100μg/kg) i.p. injection. Four hours after LPS stimulation, blood was collected by cardiac puncture and leukocyte counts performed using a Neubauer chamber. Results are expressed as a mean ± SEM of at least 5 animals per group.

**_____________________________________________________________________**


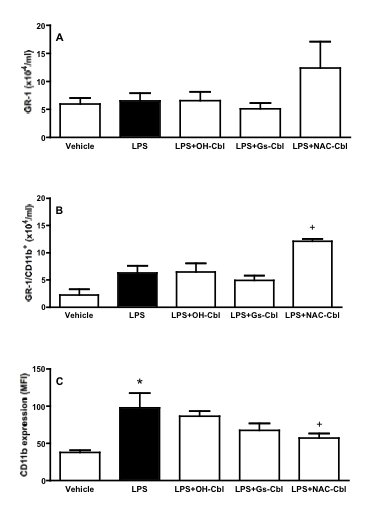


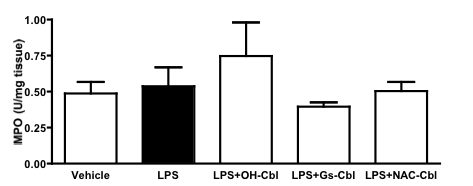


**Figure 2 - Effect of Cobalamins on CD11b expression on blood Gr1^high+^ leukocytes (PMN) during experimental endotoxaemia.** Mice were injected ip with cobalamins (200μg/kg) 1h prior and 2 h after LPS (100μg/kg) i.p. injection. Four hours after LPS stimulation, blood was collected by cardiac puncture and leukocytes stained for flow cytometry determination of CD11b expression on Gr1 cells. Results are expressed as a mean ± SEM of at least 5 animals per group. MFI = Mean fluorescence intensity.

* P<0.05 *vs.* Vehicle; + p<0.05 when compared to LPS.

**____________________________________________________________________**

**_____________________________________________________________________**


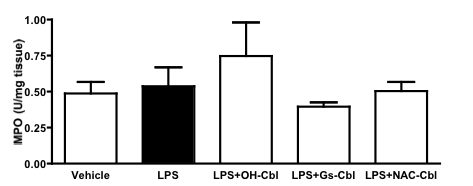


**Figure 3 - Effect of Cobalamins on neutrophil accumulation into lungs after experimental endotoxaemia.** Mice were injected i.p. with cobalamins (200μg/kg) 1h prior and 2 h after LPS (100μg/kg) i.p. injection. Four hours after LPS stimulation, lung samples were harvested and processed for myeloperoxidase activity, for assessment of neutrophil accumulation into lung tissue, as described in methods. Results are expressed as a mean ± SEM of 5 animals per group. * P<0.05 *vs*. Vehicle.

**_________________________________________________________________________________________________________________________________________**


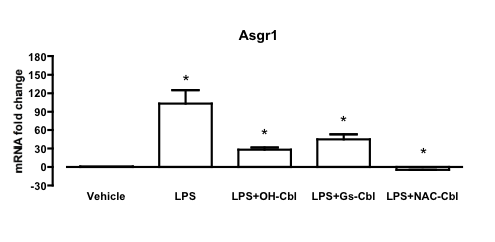


**Figure 4 - Effect of Cobalamins on Ashwell receptor/Asgr1/TC1 gene expression in liver 4h after experimental endotoxaemia.** Mice were injected i.p with cobalamins (200μg/kg) 1h prior and 2 h after LPS (100μg/kg) i.p injection. Four hours after LPS stimulation, liver samples were harvested and processed for assessment of Asgr1 gene expression (mRNA). Samples were processed and data acquired and analysed as described in methods. Results are expressed as a mean ± SEM of 3 animals per group. * P<0.05 *vs*. Vehicle

**_____________________________________________________________________**

As seen above in Figure 4, post-LPS, the endogenous HOCbl and GSCbl permit a moderate increase in expression of the hepatic receptor for the Cbl systemic carrier protein, HC1/Asgr1 (see paper scheme Figure 10), whereas the synthetic NAC-Cbl completely abrogates it.

**_____________________________________________________________________**


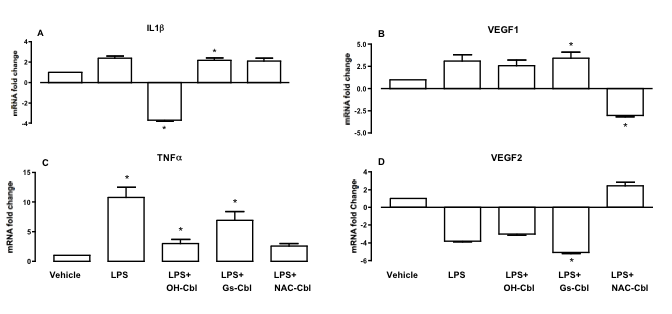


**Figure 5 - Effect of Cobalamins on gene expression of inflammation mediators in liver 4h after experimental endotoxaemia.** Mice were injected i.p. with cobalamins (200μg/kg) 1h prior and 2 h after LPS (100μg/kg) i.p. injection. Four hours after LPS stimulation, liver samples were harvested and processed for assessment of IL-1β, TNF-α, VEGF1 and VEGF2 gene expression (mRNA). Samples were processed and data acquired and analysed as described in methods. Results are expressed as a mean ± SEM of 3 animals per group. * P<0.05 *vs*. Vehicle.

**__________________________________________________________________________________________________________________________________________**


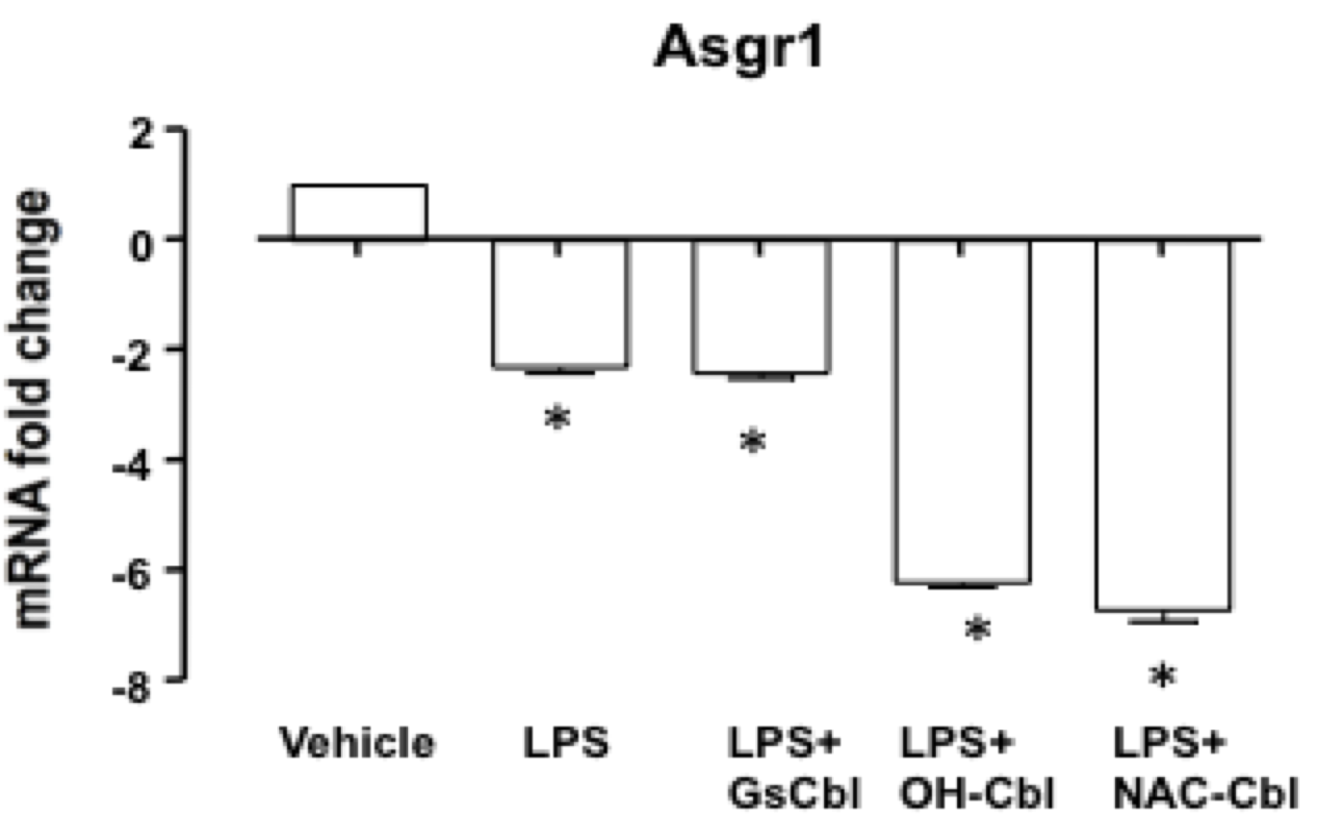


**Figure 6 - Effect of Cobalamins on Ashwell receptor Asgr1/TC1 gene expression in lungs 4h after experimental endotoxaemia.** Mice were injected with cobalamins i.p. (200μg/kg) 1h prior and 2 h after LPS (100μg/kg) i.p. injection. Four hours after LPS stimulation, lung samples were harvested, freed of blood, and processed for assessment of Asgr1 gene expression (mRNA). Samples were processed and data acquired and analysed as described in methods. Results are expressed as a mean ± SEM of 3 animals per group.

* P<0.05 *vs.* Vehicle.

**_____________________________________________________________________**


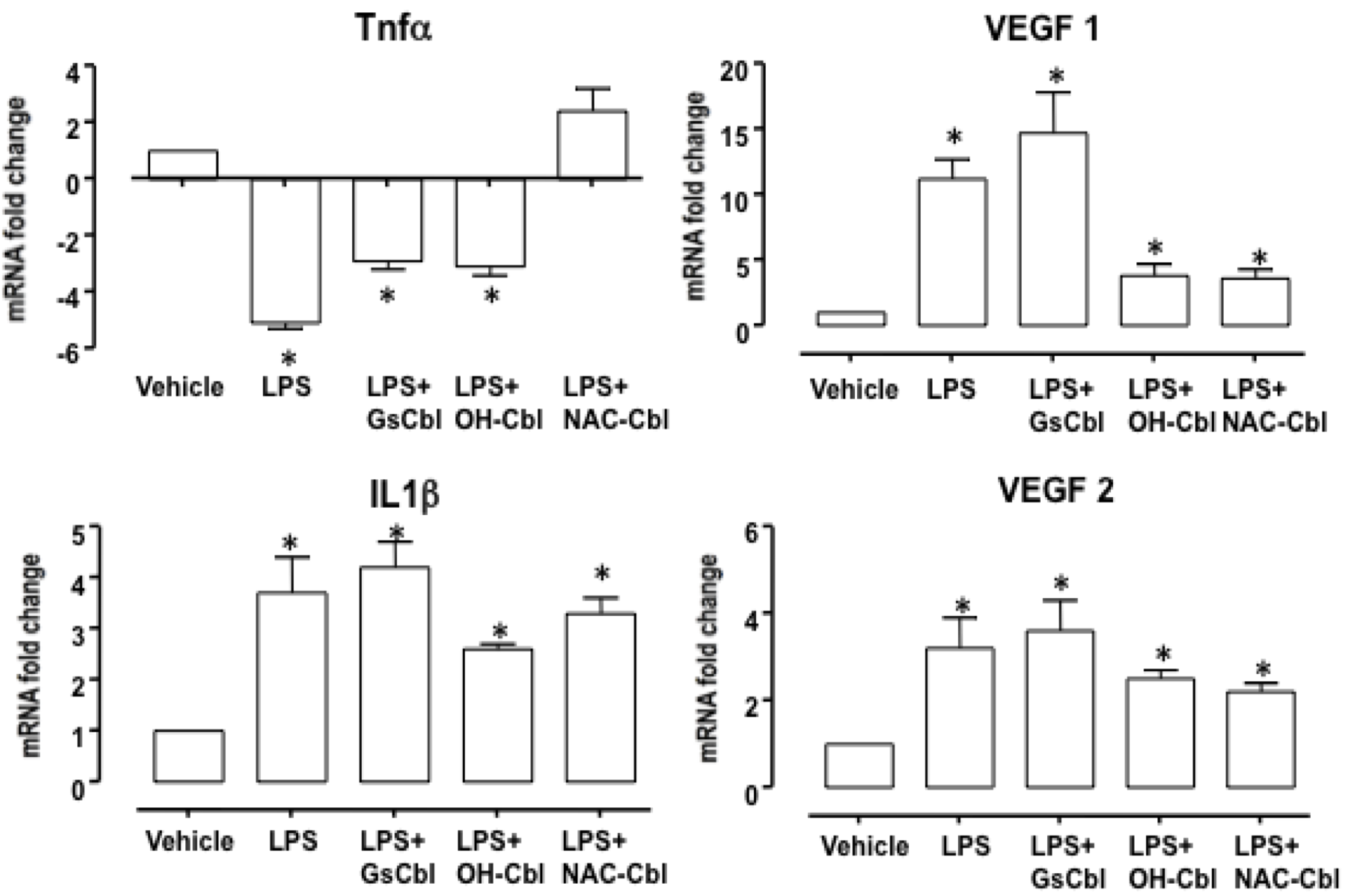


**Figure 7 - Effect of Cobalamins on gene expression of inflammation mediators in lungs 4h after experimental endotoxaemia.** Mice were injected i.p. with cobalamins (200μg/kg) 1h prior and 2 h after LPS (100μg/kg) i.p. injection. Four hours after LPS stimulation, lung samples were harvested and processed for assessment of IL-1β, TNF-α, VEGF1 and VEGF2 gene expression (mRNA). Samples were processed and data acquired and analysed as described in methods. Results are expressed as a mean ± SEM of 3 animals per group. * P<0.05 *vs.* Vehicle.

**_____________________________________________________________________**
